# Study on the Effect of Graphene Oxide with Low Oxygen Content on Portland Cement Based Composites

**DOI:** 10.3390/ma12050802

**Published:** 2019-03-08

**Authors:** Andrius Kudžma, Jelena Škamat, Rimvydas Stonys, Andrejs Krasnikovs, Denis Kuznetsov, Giedrius Girskas, Valentin Antonovič

**Affiliations:** 1 Laboratory of Composites Materials, Institute of Building Materials, Vilnius Gediminas Technical University, Saulėtekio av. 11, LT-10223 Vilnius, Lithuania; andrius.kudzma@vgtu.lt (A.K.); rimvydas.stonys@vgtu.lt (R.S.); valentin.antonovic@vgtu.lt (V.A.); 2 Laboratory of Concrete Mechanics, Institute of Mechanics, Riga Technical University, 1 Kalku street, LV-1658 Riga, Latvia; krasnikovs.andrejs@gmail.com; 3 Department of Functional Nanosystems and High-Temperature Materials, National University of Science and Technology “MISIS”, Leninsky Prospect 4, Moscow 119049, Russia; dk@misis.ru; 4 Laboratory of Concrete Technologies, Institute of Building Materials, Vilnius Gediminas Technical University, Saulėtekio av. 11, LT-10223 Vilnius, Lithuania; giedrius.girskas@vgtu.lt

**Keywords:** graphene oxide, Portland cement, hydration, fluidity, microstructure, compressive strength

## Abstract

The current study presents research into the effect of graphene oxide (GO) with a carbon to oxygen ratio of 4:1 on the fluidity, hydration, microstructure, mechanical and physical properties of Portland cement pastes and mortars. The amounts of GO investigated were 0.02%, 0.04%, and 0.06% by weight of cement, while for mortars, an extra composition with 0.1% was also prepared. According to the results, the fluidity of cement paste and mortar increased and the hydration process was slightly retarded with the addition of GO. Despite this, improvements in compressive and flexural strength were established in the mortars containing GO. The maximum effects (~22% and ~6%, respectively) were obtained with the addition of 0.06% GO. The calculation of estimated strength proportional to samples of equal density showed that for mortars cured for 7 days the gain in strength was directly related to the gain in density. For mortar samples cured for 28 days, the estimated strength was found to be significantly higher than that of the reference sample, indicating that besides density there are other factors determining the improvement in strength of mortars modified with GO. The possible structure strengthening mechanisms are discussed.

## 1. Introduction

Cement is a widely used binding material in construction. The use of cement in the production of concretes and mortars for a large number of applications has made it a very important material in civil engineering. However, cementitious materials are typically characterized as brittle materials having low tensile strength [1]. Recent developments in nanotechnology have made it possible to produce nanosized materials in the form of fibres/particles (e.g., nano-silica and carbon nanotubes) that could be used as reinforcements to prevent the microcrack initiation, and their further growth, at the outset. At the initiation stage, nano-reinforcements can control cracks at the nano scale, i.e., before they develop into micro-size cracks; therefore, in cementitious materials, they are more effective than conventional millimetre-sized reinforcements such as steel bar/fibre [2].

Based on their shape and spatial organization, nanomaterials can be classified into zero-dimensional (0D) particles, one-dimensional (1D) fibers and two-dimensional (2D) sheets [3]. The fast evolution of graphene oxide (GO) technologies has provided a new direction in modification of cement using GO nanosheets. GO has a unique two-dimensional atom-thick structure and great mechanical properties. It has been reported [4] that the intrinsic strength and Young’s modulus of GO reach 100 GPa and 1 TPa, respectively. Due to the presence of oxygen-containing functionalities (hydroxyl, carbonyl, carboxyl groups), GO exhibits good hydrophilicity and can be dispersed well in water [5]. The unique structure and high surface area of GO could be beneficial for improving the bonding between graphene sheets and cement products. The nano-sized GO with its very high specific surface area and unique two-dimensional structure could contribute to the improvement of bonds between products of cement hydration.

A review of recent publications shows that there is a lively interest in developing GO-modified structural and functional materials, including polymer- [6,7,8,9,10] and cement-based [11,12,13,14,15,16,17,18,19,20,21,22,23,24,25,26,27,28,29,30,31,32,33,34,35] composites. GO has been extensively studied in polymeric composites [6,7,8,9,10]. According to the data reported, a very small amount of GO is enough to obtain a remarkable improvement in mechanical and other properties. It has been reported [6] that the strength and toughness of GO-chitosan composites may be visibly improved with the addition of 1 wt.% GO. According to the authors, the two-dimensional geometry of GO sheets, and their wrinkled surface, provided improved nanofiller-matrix adhesion/interlocking. The elastic modulus of poly(methyl methacrylate) (PMMA) was improved by 33% with the addition of 0.01 wt.% functionalized graphene sheets (FGS) [7]. This improvement was attributed to the formation of additional hydrogen bonds between FGS hydroxyl groups, located across the FGS surfaces, and carbonyl groups of PMMA. This surface chemistry of FGS largely influences the properties of the host polymer by providing stronger interfacial interactions with PMMA.

Currently, data specifically on Portland cement-based composite modification with GO are also available in the field [25,29,36,37,38]. All the authors point out that the compressive and flexural strength of cement pastes and mortars are prone to be improved with the addition of GO. Noticeable effects are obtainable with the use of extremely low GO concentrations—from 0.02 to 1 wt.% (by weight of cement). However, there is no consensus on GO interaction with Portland cement particles and hydration products or on the particular reinforcing mechanism of Portland cement-based composites. The following models are proposed: (1) GO nanosheets regulate morphology of hydration crystals and promote formation of flower-like crystals [12]; (2) GO affects the formation of C-S-H gels and leads to a denser microstructure [36]; and (3) GO interacts with cement particles and provides the platform for C-S-H gel nucleation, resulting in the strong bond between GO and C-S-H [25,37]. The increase in strength reported varies widely: the compressive and flexural strength of cement pastes cured for 28 days was reported to increase by between 13% and 60.1%, and by between 14.2% and 90.5% respectively. Some reported results differ strongly, for example, the influence of GO on the porosity of cement pastes. A wide variation in effects can be associated with differences in experimental conditions, such as the water/binder ratio (from 0.29 to 0.5) and cement properties, as well as with features of GO used.

GO can be easily acquired from natural graphite flakes (inexpensive source) by strong oxidation and subsequent exfoliation. Several methods for producing GO are known [38]. In the above-mentioned studies, GO produced by a modified Hummers method was mainly used for the modification of Portland cement-based composites. Highly oxidized GO produced in this way typically has C to O ratio of 2:1 [38]. As shown in [39], the properties of GO change strongly with the increase in C:O ratio, i.e., with the decrease in GO oxidation level. It is widely believed that the efficiency of GO in Portland cement-based composites increases with the increase in its oxidation level, due to the presence of more oxygen-containing functional groups. However, according to the results published by Murugan et al. [29], the improvement in mechanical properties of cement pastes can be also obtained with the use of reduced GO (rGO), where a majority of oxygenated groups are removed during the post reduction process. From this point of view, some commercially available GO products, in which oxygen content is less than that in highly oxidized GO, may also exhibit particular potential for application in cement-based composites. Such type of GO products are now commercially available; however, the effect of low-oxidized GO additives on the properties of Portland cement-based composites has not been adequately studied. The lack of information in this field restricts GO implementation as a valuable additive in the production of building materials and structures.

The current study focused on the investigation of the properties of cement pastes and mortars modified with low oxidized GO, in which the C to O ratio is around 4. The study included characterization of the GO used, investigation of fluidity, hydration and hardening of cement pastes, and analyses of microstructure and phase composition of hardened cement pastes, along with determination of the physical and mechanical properties of hardened mortars. To more accurately determine the effect of GO additive on the process of Portland cement hydration and formation of cement stone structure, cement paste was investigated. Physical and mechanical properties are more relevant for the final product—mortar or concrete. Therefore, mortars were investigated to establish the effect of GO on their density and strength.

## 2. Materials and Methods

### 2.1. Materials

The cement paste and mortar investigated in this study were prepared using CEM I 42.5R Portland cement (SC Akmenės cementas, N. Akmenė, Lithuania) and GO dispersion with a solid content of 500 mg/L. The chemical and physical properties of the cement are presented in Table 1 and Table 2. Sand of particle size 0/4 according to EN 13139 [40] was used (Table 3). Polycarboxylate superplasticiser (Sp) with a solid content of 27% was used (pH, 5–7; density, 1.07 kg/dm^3^; maximum chloride content <0.10 wt.%; maximum alkali content <1.5 wt.%). Commercially available nano graphene oxide suspension from the “Graphene Supermarket” was used. The specifications of GO provided by the producer are presented in Table 4.

### 2.2. Preparation

#### 2.2.1. Preparation of the Cement Paste

Cement pastes with different amounts of GO (0%, 0.02%, 0.04%, and 0.06%) and with Sp were investigated. The amount of Sp was 0.5% by weight of cement, and the water to cement ratio (W/C) was 0.27. Pastes without Sp were also investigated to evaluate the impact of GO on rheological properties. W/C in these pastes was 0.30. The codes of cement paste samples and quantities of materials are given in Table 5. The mixing procedure for the pastes was in accordance with EN 196-3 [42]. The GO suspension was mixed with water and plasticizer (when used); then it was dispersed for 2 min with an ultrasonic device (UZDN-2T, UkrRosPribor, Belgorod, Russia), which works at 44 kHz frequency. Then, the dispersion obtained was added into the cement and mixed for 3 min (1 min at low speed (62 ± 5 min^−1^) and 2 min at high speed (125 ± 10 min^−1^)). Before mixing with water, the cement was dry mixed for 1 min at low speed with a planetary movement mixer.

#### 2.2.2. Preparation of the Mortar

The plasticized mortar consisting of 3 parts sand and 1 part cement (by weight) was investigated. The water to cement ratio (W/C) in mortar was 0.5, amount of Sp—0.5% by weight of cement, amount of GO—0%, 0.02%, 0.04%, 0.06%, and 0.10% by weight of cement. The mixing procedure, moulding and curing of samples (40 mm × 40 mm × 160 mm) was in accordance with EN 196-1 [42] requirements. The GO suspension was mixed with water and plasticizer, then it was dispersed for 2 min with an ultrasonic device (UZDN-2T), which works at 44 kHz frequency. Next, the dispersion obtained was added into the dry mixture of cement and sand and mixed for 3 min (1 min at low speed (62 ± 5 min^−1^) and 2 min at high speed (125 ± 10 min^−1^)). Before mixing with water, the mixture of cement and sand was dry mixed for 1 min at low speed with a planetary movement mixer. The codes of samples and quantities of materials are listed in Table 5.

### 2.3. Testing

#### 2.3.1. FTIR and Raman Spectroscopy

FTIR spectra of GO were recorded in transmission mode on an ALPHA FTIR spectrometer (Bruker, Inc., Ettlingen, Germany) equipped with a room temperature detector DLATGS (Bruker, Inc., Ettlingen, Germany). The spectral resolution was set at 4 cm^−1^. Spectra were acquired from 40 interferogram scans. The sample was prepared by the dropping of 250 μL aqueous GO suspension on a Si plate and subsequent drying of the layer. A bare Si plate was used for the collection of a background spectrum. Parameters of the bands were determined by fitting the experimental spectra with Gaussian-Lorentzian shape components using GRAMS/A1 8.0 (Thermo Fisher Scientific, Waltham, MA, USA) software. Raman spectroscopy measurements were performed using a DXR Raman microscope (Thermo Fisher Scientific, Waltham, MA, USA) with excitation from a laser beam (532 nm) at low power level (1 mW) in order to avoid damaging the organic functional groups. The suspension was placed on a glass plate and air-dried for 20 min.

#### 2.3.2. Fluidity of the Cement Paste/Mortar

The fluidity of non-plasticized cement pastes and plasticized cement pastes and mortars just after mixing was determined in accordance with EN 1015-3 [43]. A flow table test method was applied and the measurements were performed with 1 mm accuracy. The mean values of three individual measurements are provided along with standard deviations.

#### 2.3.3. Hydration Temperature of Cement Paste

The temperature of the exothermic reaction (EXO) during the hydration and hardening of cement pastes was determined according to the Alcoa Industrial Chemicals test method [44]. Just after mixing, the cement pastes were cast in the moulds under light vibration (10 s). The weight of the cast sample (EXO specimen) was approximately 300 g. The filled mould was placed into the insulating box and a thermocouple was placed into the freshly mixed composition. The variation in temperature inside the mix was recorded, along with the time of measurement, using the data logger connected to the thermocouple and personal computer.

#### 2.3.4. Ultrasonic Pulse Velocity (UPV) in Cement Pastes and Hardened Mortars

The Pundit 7 (Schleibinger Geräte GmbH, Buchbach, Germany) device using two 54-kHz standard cylindrical transducers (transmitter and receiver) measured the UPV of cement pastes and mortars. The transducers were pressed against the samples at two precisely opposite points. The UPV in m/s was calculated from the equation (*S*/*t*) × 10^6^, where *S* is distance in meters and *t* is time in microseconds.

#### 2.3.5. Microstructural and X-ray Diffraction Analyses

For a qualitative phase analysis of cement paste, an X-ray diffractometer “DRON-7” (Bourevestnik, St. Peterburg, Russia) with graphite-monochromatized Cu-Kα (λ = 0.1541837 nm) radiation was used. The test parameters were: 30 kV voltage; 12 mA current; diffraction angle 2θ range from 4° to 60°, detector movement step 0.02°; exposure time per step 0.5 s. Phase identification was carried out by decrypting XRD patterns with the use of PDF-2 (2003) and ICDD diffraction data bases. The variation in the quantity of the obtained phases was evaluated through the measurement and comparison of the intensity of the main diffraction peaks. The microstructural analysis was performed using an SEM JEOL JSM-7600F (JEOL Ltd., Tokyo, Japan) scanning electron microscope (SEM) coupled with an energy dispersive spectrometer (EDS) IncaEnergy 350 (Oxford Instruments) for X-ray microanalysis. The cleavage surface of hardened cement paste, pre-coated with a current-conducting layer of gold in a QUORUM Q150R ES (Quorum technologies Ltd., Laughton, UK) vacuum sputter coater, was investigated. For the analysis of morphology and elemental composition of GO, a few ml of GO suspension were dropped on a pad, dried in the exicator at room temperature and then pre-coated with a current-conducting layer of gold.

#### 2.3.6. Mechanical Properties of Mortars

Flexural strength and compressive strength of the hardened mortars cured for 7 and 28 days, respectively, were determined in accordance with EN 196-1 [41]. After prepared mortar mixture was cast into the moulds, the samples were covered with glass plates and cured for 1 day in the moulds at humidity of not less than 50%. Then, the samples were demoulded, submerged in potable water and cured there at 20 ± 1 °C temperature for 6 or 27 days. The samples were tested not more than 4 h after they were taken out from the water bath. The Tinius Olsen H200Ku-0032 testing machine (Tinius Olsen Ltd., Salfords, UK) was used, equipped with shear web type load cell, having 250 kN capacity and providing the accuracy of 0.5% of applied force. For the flexural strength testing with three points, the loading rate was 50 N/s. Two supporting rollers were spaced 100 ± 5 mm apart and a third steel loading roller was placed centrally between other two. For the compressive strength testing, the loading rate was 2400 N/s.

#### 2.3.7. Estimated Strength of Mortars

The actual volume density of samples modified with various GO dosages differed. To evaluate the effect of density variation on the change in strength, the obtained actual compressive and flexural strength values were recalculated to “estimated strength” *f_s_* of mortar samples with volume density selected as follows: 2165 kg/m^3^ for mortars cured for 7 days and 2222 kg/m^3^ for mortars cured for 28 days. These density values corresponded to the reference mortar samples GOM0 cured for 7 and 28 days, respectively. The following formula was used [45]:

(1) $$f_{s}=f_{a}\cdot \rho_{s}^{2}/\rho_{a}^{2}$$

where *f_s_* is the strength of the sample with selected volume density, *f_a_* is the strength of GOM sample of actual volume density, *ρ_s_* is the selected volume density, and *ρ_a_* is the actual volume density of GOM sample.

## 3. Results and Discussion

### 3.1. GO Characterization

**Figure 1:**
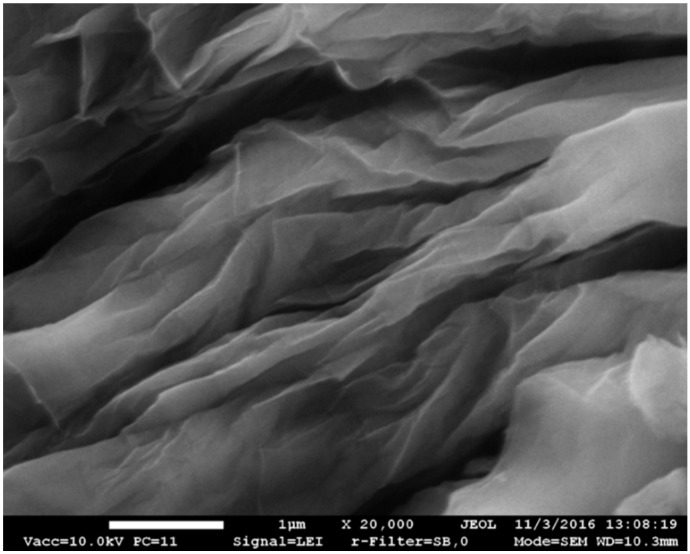
Scanning electron microscope (SEM) image of undispersed GO sheets.

Figure 1 shows the general view of undispersed GO sheets obtained after drying of the GO suspension. The results of X-ray microanalysis by EDS showed that GO consisted of ~77 wt.% C and ~19 wt.% O. The remaining ~4% included technological residues such as Mn (~3%), K, Cl, S (most probably, technological residues of GO preparation) and such elements as Si, Ca, Al, Cu, Zn (most likely, residues of primary graphite impurities). According to EDS results, the actual C/O ratio was 4.05.

**Figure 2:**
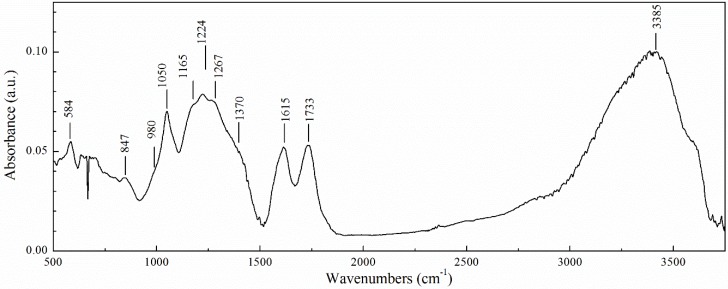
Fourier transform infrared (FTIR) spectra of GO.

Figure 2 presents the Fourier transform infrared (FTIR) spectra of GO. According to the data reported [12,46,47,48], the main bands that appeared at the spectra were identified as follows. The broad absorption band between ~3000 cm^−1^ and ~3700 cm^−1^ wavenumbers with the maximum at ~3385 cm^−1^ is associated with the O–H bonds stretching. The absorption band at 1733 cm^−1^ is typical for C=O bond stretching in carbonyl moieties and carboxyl groups that are located mostly at the edges of the GO sheet but also on the basal planes. The band at 1615 cm^−1^ most likely appeared due to both the vibration of double C=C bonds and deformation of water molecules (H–O–H bend). The two bands at 1224 cm^−1^ and 1050 cm^−1^ may be associated with the C–O–C and C–O, respectively. The band at 1165 cm^−1^ is associated with C–OH bond stretching. The band at 847 cm^−1^ may be attributed to C–O–C stretching vibration of the epoxy ring [48]. The weakly expressed bands at ~1370 cm^−1^, ~1267 cm^−1^ and ~980 cm^−1^ may testify to the presence of sulfur containing groups (–C–SO_3_^−^). Thus, FTIR results confirmed the presence of functional oxygen containing epoxyl, carboxyl, carbonyl, hydroxyl groups, typical for GO prepared using the modified Hummers method.

**Figure 3:**
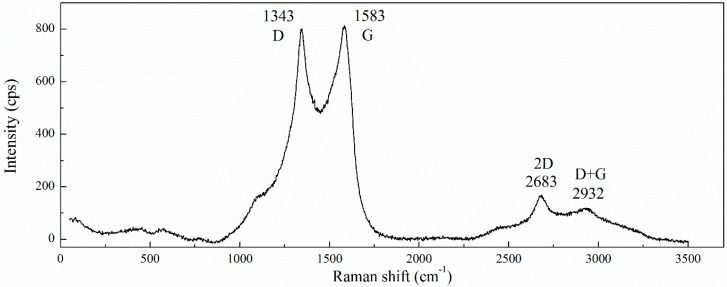
Raman spectra of GO.

The results of Raman spectroscopy are presented in Figure 3. The main features typical for Raman spectra of graphitic carbon-based materials can be observed in the spectra obtained. The first-order so-called G band comes from the stretching of the C–C bond in graphitic materials, and is common to all sp^2^ carbon systems [49,50]. As reported in the literature [49], for pristine graphite G the band occurs at around 1580 cm^−1^. During oxidation, oxygen-containing functional groups are attached to the basal plane of graphite sheets resulting in lattice distortion. This typically causes the G peak to shift to a higher Raman frequencies range of ~1590–1610 cm^−1^ [50,51,52]. In our case, the G band appeared at around 1583 cm^−1^ and that is much lower compared to highly-oxidized graphene and is very close to pristine graphite; this can be related to the low oxidation level of GO (C:O is ~4). The D and 2D bands originate from the second-order double and triple resonant processes, respectively [53]. The D band appeared at around 1343 cm^−1^. Since the D band is caused by the disordered structure of graphene due to attached oxygen functionalities, the ratio of peak intensities *I_D_*/*I_G_* is often used to characterize the level of disorder in graphene materials [49]. The *I_D_*/*I_G_* ratio obtained from Figure 3 was ~0.98, which is comparable to those reported in other sources for GO. The 2D band appeared at ~2683 cm^−1^. The shift and shape of the 2D overtone was correlated with the number of graphene layers [47]. As shown in [49], the sharp and intensive 2D peak is typical for single-layer graphene and the ratio of the intensities of the 2D and G peaks, *I_2D_*/*I_G_*, continuously decreases with increasing graphene layer number. The low-intensive broad 2D band obtained in the present study may testify that a part of GO in the analysed suspension is in the form of multi-layer nanoparticles. A D+G disorder peak is also observable at 2932 cm^−1^.

### 3.2. Influence of GO on the Properties of Cement Pastes and Mortars

#### 3.2.1. Influence of GO Addition on the Fluidity of the Cement Paste/Mortar

The consistency of mortar is one of the main properties determining the ease with which it can be mixed, transported, placed and compacted to give a uniform product. The results showing the impact of GO on the fluidity of cement paste and mortar are presented in Table 6 and Table 7. In all cases an increase in fluidity was observed with the addition of GO. For cement paste without Sp (GO0, GO2, GO4, and GO6), the fluidity of all pastes with GO increased by ~4% and no dependence on GO dosage was observed. For plasticized cement paste and mortar, an analogous insignificant ~4–7% fluidity increment in samples with GO was observed; however, the gradual increase in fluidity with the increase in GO dosage should be pointed out. It is noticeable also, that with the addition of GO from 0.02% to 0.06% it is possible to reach the same fluidity of cement paste as with the addition of 0.5% Sp. The obtained dependence is the reverse of that reported in earlier works. According to earlier reported results [25,26], modification of ordinary Portland Cement (OPC) with a small dosage (0.02–0.04%) of highly oxidized GO (C:O = 1:2.3) leads to a significant reduction in cement paste fluidity. Two main hypotheses were proposed to explain this effect. According to the first hypothesis [20], due to hydrophilicity and large surface area GO is able to absorb water molecules on its surface, thus reducing the free water content and leading to the decrease of fluidity. According to Shang et al. [25], the negatively charged GO interacts with cement particles by electrostatic interactions, causing flocculation that entraps a large amount of free water and, therefore, plays a decisive role for the rheological behaviour of cement pastes. Wang et al. [24] reported the increase of fluidity; however, only when cement compositions contained 10% and 20% fly ash, which is characterized by less water demand and acts like a bouncing “ball”. The results of the current research showed that in the case of low oxidized GO, the interaction between GO and cement particles may be run by other mechanisms. A possible mechanism is proposed in Section 3.2.4.

#### 3.2.2. Influence of GO Addition on the Hydration Heat of Cement

**Figure 4:**
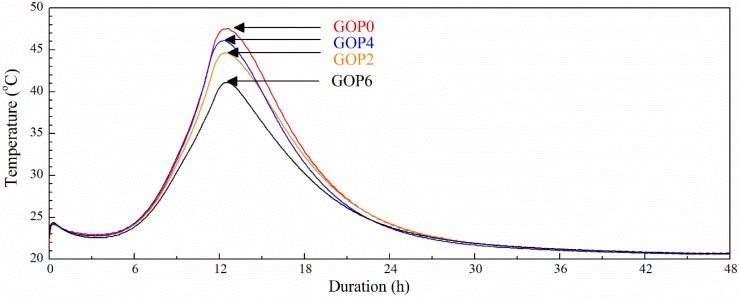
Exothermic reaction (EXO) temperature as a function of time for plasticized cement pastes.

Figure 4 shows heat developing curves for GO-modified cement pastes with Sp. All the curves obtained exhibited five typical hydration periods [54]: the initial reaction associated with the wetting of cement particles and C_3_S dissolution; the induction period, when the dissolution rate of C_3_S decreases and C-S-H gel forms; the acceleratory period associated with the precipitation of C-S-H gel and CH; the deceleratory period; and a period of slow reaction. During the initial reaction, the temperature of the GO-unmodified cement paste sample (GOP0) reached ~24 °C. The induction period lasted around 4 h and was followed by the acceleration period. The maximum temperature of the hydration process for the GOP0 sample reached ~48 °C after ~12.5 h. With the addition of 0.02%, 0.04% and 0.06% of GO, respectively, the temperature of the initial reaction did not change and the peak temperature of hydration was reached after the same time. However, the maximum process temperature decreased to ~44 °C for GOP2, ~46 °C for sample GOP4 and ~41 °C for sample GOP6, indicating that the hydration process was slightly retarded with the addition of GO. Reduction of the total amount of the heat released during Portland cement paste hydration with the increase of GO additions was also reported by Wang et al. [55].

#### 3.2.3. Influence of GO Addition on the Compactness of Cement Pastes

**Figure 5:**
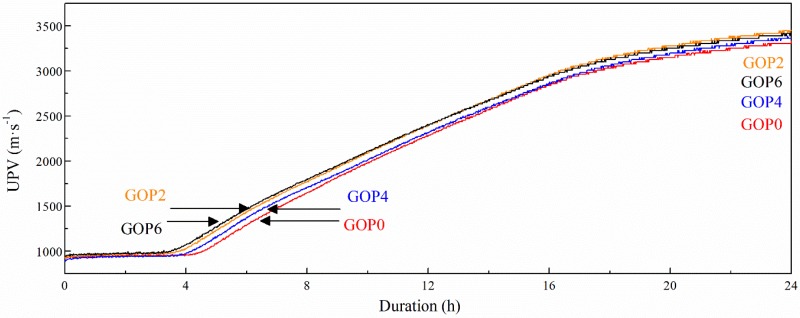
Ultrasonic Pulse Velocity (UPV) as a function of time for the plasticized cement pastes.

Figure 5 shows ultrasonic pulse velocity in plasticized cement pastes over time. At the initial stage, before the start of the paste setting, UPV in all the pastes was around 980 m·s^−1^. Then the rise of UPV was observed, related to the setting of the pastes and the growth of their density. GO addition evidently shortened the setting time of cement pates, i.e., accelerated paste setting and structure compaction. The setting time of the reference sample GOP0 was the longest(~4.5 h). GOP6 containing 0.06% GO, showed the shortest setting time(~3.5 h). After 24 h of measurements, the UPV in all GO-modified pastes was higher than that in the reference, indicating the formation of a more compacted and solid structure. However, no direct dependence between GO dosage and increase in UPV was noted. The samples containing 0.02% and 0.06% GO showed very similar results and the highest UPV (3450 m·s^−1^ and 3420 m·s^−1^, respectively), while 0.04% GO dosage corresponded to the medium UPV value (3350 m·s^−1^). UPV in GOP0 after 24 h was 3300 m·s^−1^.

#### 3.2.4. The Effect of GO on Fluidity and Hydration of Pastes

The fluidity test, along with the measurements of EXO temperature and UPV, showed that GO had a complex influence on the hydration of cement pastes: two or more competitive effects may take place during hydration of cement pastes modified with low oxidized GO. The fluidity of GO-modified cement pastes (determined just after mixing) was found to be increased, indicating that at the early stage (just after mixing) GO may act as an electrostatic dispersant for cement particles—similar to a superplasticizer. Since for low oxidized GO the number of functionalities is much less as compared with highly oxidized GO, its surface has much less negative charge. This may cause the domination of electrostatic repulsion and steric effects just after mixing. It is well known also, that in dispersions the charge of GO is pH-dependent; the higher pH the more functional groups are ionized to form negatively charged radicals [56]. The pH of fresh cement paste tends to increase over time; as a result, the charge of GO may become more negative. Therefore, with time, other mechanisms may be switched on, according to which GO sheets containing a particular amount of functional oxygen groups act as additional nucleation sites and speed up crystallization of hydrates and setting of cement paste, as is confirmed by the shorter setting time for samples containing GO. A decreased EXO temperature normally indicates retarded hydration,however, further investigation is required to detail the mechanism.

#### 3.2.5. Influence of GO Addition on the Phase Composition and Microstructure of Hardened Cement Pastes

**Figure 6:**
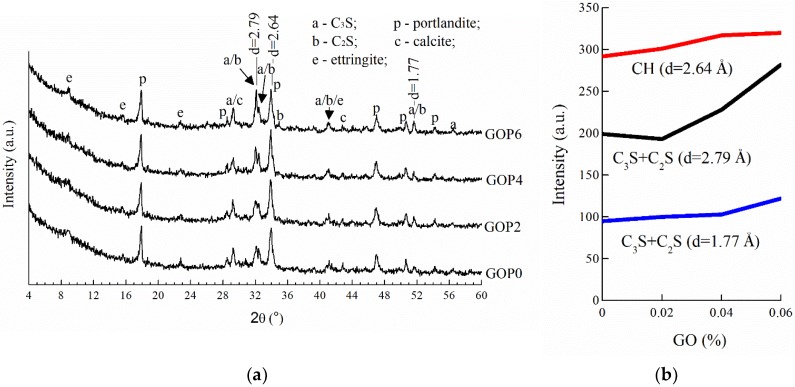
XRD patterns of cement pastes cured for 28 days (**a**), and intensities of main CH, C_3_S and C_2_S peaks as a function of GO dosage (**b**).

Figure 6a presents XRD patterns of the GO-modified cement pastes (GOP2, GOP4, GOP6) and the reference sample (GOP0) cured for 28 days. The main reflections appearing in all patterns are attributable to typical crystalline products of Portland cement hydration (portlandite CH or Ca(OH)_2_ and ettringite C_3_AS_3_H_32_) and un-hydrated cement silicates (alite C_3_S and belite C_2_S). Low-expressed convexity of diffraction curves between ~27° and ~35° 2-theta is associated with the presence of amorphous C-S-H gel—the main product of OPC hydration, whose study with X-ray diffractometry is limited. As can be seen from the patterns presented, the modification of cement paste with GO did not cause any observable changes in its phase composition. This observation is consistent with the results reported earlier [29,57]. However, a slight variation of the intensities of main peaks, attributable to CH (d = 2.64 Å) and cement silicates (d = 2.79 Å), can be observed. Thus, with the addition of 0.02%, 0.04% and 0.06% GO, the intensity of the main CH peak increased by 4%, 10% and 12%, respectively (Figure 6b). Wang et al. [57] and Gong et al. [58] also reported on the increase in CH amount and CH crystal size with the addition of 0.02%–0.08% GO in their work. The silicates peak intensity did not change visibly with the addition of 0.02% GO, and then increased by 15% and 42% with the addition of 0.04% and 0.06% GO, respectively. A similar trend was observed in intensity variation of other silicate peaks (d = 1.77 Å), indicating that the amount of un-hydrated cement silicates was increased.

**Figure 7:**
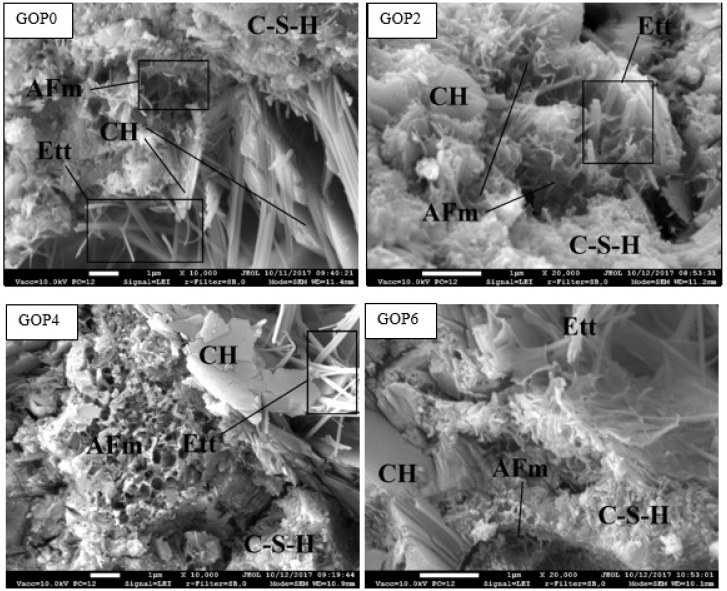
Microstructure of plasticized cement pastes cured for 28 days: AFm—tricalcium aluminate monosulfate hydrate; CH—portlandite (calcium hydroxide); C-S-H—calcium silicate hydrate; Ett—ettringite (calcium trisulfoaliuminate hydrate).

It is well known that the microstructure of structural materials largely determines their mechanical properties. According to the results presented by other researchers [12,55], GO addition has a significant impact on the microstructure of cement pastes and crystal morphology changes strongly with increasing GO dosage. It is reported that GO regulates the cement hydration process by forming polyhedron and flower-like crystals depending on the GO amount, and thus the mechanical properties of cement composites are determined largely by the crystals’ morphology. Murugan et al. [29] found that when cement was modified with 0.02% reduced graphene oxide, thin non-uniform platelets and a network of rod-like crystals are formed in the structure of the cement paste cured for 28 days, causing the increase in compressive and flexural strength. In the current experiments, to evaluate the effect of GO (with C to O ratio equal ~4) on the microstructural evolution in Portland cement paste, microscopic analysis was performed for GOP series cement pastes cured for 1, 3, 7, 14 and 28 days. In GO modified pastes, no evidence of flower-like or polyhedron crystal formation was found, and there were no any other visible changes in microstructure morphology either, as compared with the reference sample GOP0. The structures of cement pastes cured for 28 days, observed from the cleavage surface under high magnification, are shown in Figure 7. The main microstructure features identifiable from their typical morphology were as follows: portlandite platelets, ettringite needles, calcium silicate hydrates (C-S-H), and tricalcium aluminate monosulfate hydrates (AFm). The remaining part of hardened cement paste represented mostly compact material without specific structural elements. The surface of the hardened cement paste pores was covered with hexagonal CH platelets and ettringite needles. The microstructure of hardened cement paste modified with GO did not differ visibly from that of the reference sample.

#### 3.2.6. Influence of GO Addition on the Strength and Density of the Hardened Mortars

**Figure 8:**
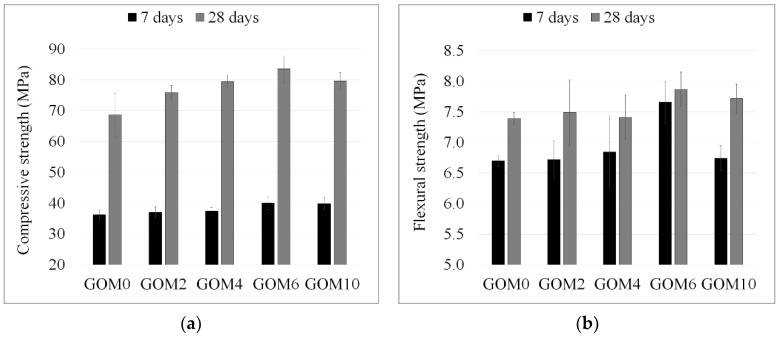
Compressive (**a**) and flexural (**b**) strength of mortars cured for 7 and 28 days, respectively.

Figure 8a presents the compressive strength of GO modified mortars cured for 7 and 28 days, respectively. After 7 days, the influence of GO on the strength of mortars containing 0.02% and 0.04% GO was negligible. When 0.06% GO was added, the compressive strength increased by 10.5%. The more visible effects were observed after 28 days curing. The strength of all GO-modified mortars was found to increase as compared to the reference sample. The gradual rise in compressive strength (by 10.5–21.7%) was observed with the increase in GO dosages from 0.02% to 0.06%. The maximum effect (21.7%) was obtained with 0.06% GO. Whereas a rising strength trend was established in the GO range up to 0.06%, the additional composition of mortar with 0.1% GO (GOM10) was prepared. A similar ~10% compressive strength improvement effect was obtained after 7 days curing of the mortar containing 0.1% GO (GOM10); however, after 28 days the gain in compressive strength was lower compared to the mortar with 0.6% GO. The effect of GO on the flexural strength of mortars was expressed to a lesser extent (Figure 8b). A slight increase in flexural strength can be noted only with the addition of 0.06% GO; after seven days, the increase in flexural strength was 14.3%, however, in more aged mortars this effect was reduced and only a 3% difference between the GO-modified and the control sample was observed.

**Figure 9:**
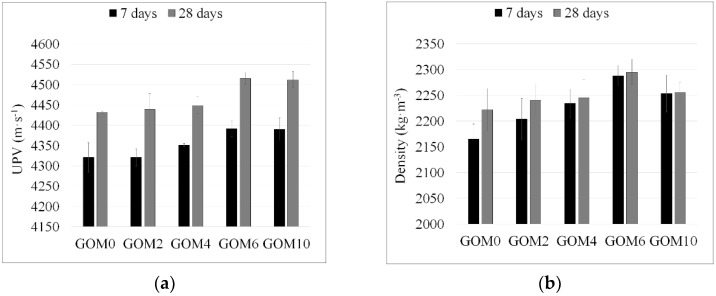
Dry bulk density (**a**) and UPV (**b**) of mortars cured for 7 and 28 days, respectively.

Dry bulk density of GO modified mortars was determined after 7 and 28 days curing, respectively (Figure 9a). The density of the reference sample GOM0 cured for 7 days was 2165 kg∙m^−3^. With the addition of 0.02%, 0.04% and 0.06% GO a gradual increase in mortar density up to 2204, 2234 and 2288 kg∙m^−3^, respectively, was observed; with a further increase in GO dosage (0.1%), the density decreased. A similar GO effect was obtained on mortar samples cured for 28 days—the sample containing 0.06% GO showed the maximum density. However, the greater the GO dosage in mortar was, the lesser was the density increment in samples cured for 28 days, compared to those cured for 7 days. Finally, for sample GOP6, the density after 28 days differed a little from the density after 7 days, indicating that GO modification has much more influence on the structure compaction in early hydration and hardening periods.

UPV in materials depend both on the structural continuity and the density of the material. Ultrasonic wave flows faster in solids with lower actual density; at the same time, the structural discontinuity (pores, cavities, cracks, etc.) disturbs ultrasonic pulse (UP) propagation and decreases its velocity. Moreover, ultrasonic waves travel more slowly in crystalline structures compared with amorphous ones [59]. UP velocity measurement results (Figure 9b) revealed that UPV gradually increased with GO dosage and reached the maximum at 0.06% GO, indicating a strong correlation with the results of density measurements and mechanical properties of mortars. In consideration of the above-mentioned dependencies between UPV and structure features in the current study, the increase in UPV is most likely to be associated with three factors: structure compaction, reduced porosity and increase in C-S-H gel volume. However, the results of X-ray diffractometry and exotherm profiles obtained showed that the hydration process was slightly retarded with the addition of GO and the amount of un-hydrated silicates (C_3_S, C_2_S) increased. At the same time, the volume of crystalline hydration products (CH) slightly increased. Therefore, it is unlikely that more C-S-H gel formed in GO-modified cement pastes. Thus, the increase in the paste density and UPV may be mostly associated with the first two factors. The reduction of the cumulative pore volume in hardened cement paste with the addition of GO was also reported by Wang et al. [55] and Murugan et al. [29].

To evaluate the density increase effect on the mortar strength, the obtained strength values were recalculated into the strength *f_s_*, corresponding to one selected density (Table 8). The estimated strength of GO modified mortars cured for 7 days was found to differ insignificantly from the reference sample—the variation of *f_s_* values was around ±1 MPa, allowing to assume that the increase in mortar strength after seven days curing may be directly related to the increase in density. For the mortars cured for 28 days, the values of estimated strength showed a rising trend with the increase in GO dosage up to 0.06%, indicating that, besides density, there are other factors determining the improvement in mechanical properties.

Several strengthening mechanisms, non-related to the change in crystal morphology, proposed in previous studies, seem to be possible: for example, formation of 3D network as a result of chemical reaction between –COOH groups on the edge of GO sheets and Ca^2+^ of Ca(OH)_2_ in cement system [35]; development of strong bonds with C-S-H during nucleation due to supplying reactive sites by functional groups [20]; and control of cracks due to better mechanical interlocking and strong interaction between the cracks and GO sheets [11].

## 4. Conclusions

In the current experiment, nano graphene oxide with a carbon to oxygen ratio equal ~4, having all functional groups (epoxyl, carboxyl, carbonyl, hydroxyl) typical for GO and prepared with a modified Hummers method, was used for the modification of OPC pastes and mortars. According to the results obtained, the modification of OPC with such a type of GO increases the fluidity of cement paste and mortar just after mixing: the effect of 0.02–0.06% GO on the fluidity of cement paste is comparable with the effect of 0.5% of polycarboxylate superplasticizer. At the same time, the setting time of GO-modified cement pastes was 0.5–1.0 h shorter than that of control paste. The decrease in maximum hydration temperature and the increase in silicate (C_3_S + C_2_S) peak intensities in XRD patterns testified that the hydration process was slightly retarded with the addition of GO to cement pastes. All the above-mentioned results testify that two or more competing effects may be provoked by GO in the hydration of Portland cement system. Despite this, the improvement in compressive strength (and less expressed influence on flexural strength) of GO modified mortars was established in the samples containing 0.02–0.1% GO. The maximum effect was obtained with the addition of 0.06% GO: the compressive strength of the sample containing 0.06% GO was 83.5 MPa, the flexural strength was 7.9 MPa and this is ~22% and ~6%, respectively, higher than those of the reference sample. Since no changes in microstructure morphology and phase composition were observed, the improvement of mechanical properties may be mainly associated with the structure compaction (reduction in microstructure discontinuity and porosity), as confirmed by the measurements of density and UPV. The calculation of estimated strength in proportion to equal density of samples showed that the gain of strength was directly related to the gain of density only for mortars cured for seven days. For mortar samples cured for 28 days, the estimated strength was found to be significantly higher than that of the reference sample, indicating that, besides density, there are other factors determining the improvement of mechanical properties.

## Figures and Tables

**Table 1:** Chemical composition of cement, %.

CaO	SiO_2_	Al_2_O_3_	Fe_2_O_3_	MgO	K_2_O	Na_2_O	SO_3_	Other
62.46	19.23	4.91	3.50	3.19	0.94	0.12	3.10	2.55

**Table 2:** Physical and mechanical properties of cement (data provided by producer).

Properties	Portland Cement CEM I 42.5 R
Specific surface, m^2^/kg	379
Standard consistency, %	27.4
Initial setting time, min	147
Final setting time, min	204
Compressive strength after 7 days, MPa	31.4 *
Compressive strength after 28 days, MPa	53.8 *

* determined in accordance with standard EN 196-1 [41].

**Table 3:** The granulometric composition and the bulk density of 0/4 sand.

Raw Material	Passing by Sieve Opening Size, %								Bulk Density, kg/m^3^
	0.063	0.125	0.25	0.5	1	2	4	5.6	
**Sand (fraction 0/4)**	0.17	0.93	10.01	41.17	69.91	90.39	99.45	99.98	1620

**Table 4:** Specification of graphene oxide (GO) (data provided by GO producer).

Properties	Description
Composition	Carbon (79%), Oxygen (20%)
Thickness	1 atomic layer (about 60% of material)
Flake size	0.5–5 micron
Suspension	500 mg/L

**Table 5:** Codes of cement paste and mortar samples.

Samples	Codes	Cement, g	Sand, g	GO, % *	Sp, % *	Water/Cement
Cement pastes without Sp	GO0	3600	-	0	-	0.30
	GO2	3600	-	0.02	-	0.30
	GO4	3600	-	0.04	-	0.30
	GO6	3600	-	0.06	-	0.30
Cement pastes with Sp	GOP0	3600	-	0	0.50	0.27
	GOP2	3600	-	0.02	0.50	0.27
	GOP4	3600	-	0.04	0.50	0.27
	GOP6	3600	-	0.06	0.50	0.27
Mortars with Sp	GOM0	900	2700	0	0.50	0.50
	GOM2	900	2700	0.02	0.50	0.50
	GOM4	900	2700	0.04	0.50	0.50
	GOM6	900	2700	0.06	0.50	0.50
	GOM10	900	2700	0.10	0.50	0.50

* % by weight of cement.

**Table 6:** Cement paste fluidity.

Paste	Without Sp				With Sp			
	GO0	GO2	GO4	GO6	GOP0	GOP2	GOP4	GOP6
Fluidity, mm	165 ± 1.3	172 ± 1.0	171 ± 0.8	172 ± 0.8	171 ± 1.8	175 ± 1.0	177 ± 0.8	178 ± 1.3

**Table 7:** Mortar fluidity.

Mortar	GOM0	GOM2	GOM4	GOM6	GOM10
Fluidity, mm	195 ± 2.0	200 ± 2.5	205 ± 2.3	205 ± 2.0	208 ± 2.3

**Table 8:** Estimated compressive strength *f_s_* of mortars, MPa.

Samples	7 Days	28 Days
GOM0	36.2	68.6
GOM2	35.7	74.6
GOM4	35.1	77.8
GOM6	35.8	80.1
GOM10	36.7	77.3
